# Microstructure and interaction in aluminum hydrides@polydopamine composites and interfacial improvement with GAP adhesive

**DOI:** 10.1038/s41598-024-59944-1

**Published:** 2024-05-01

**Authors:** Qi An, Wuxi Xie, Yajin Li, Xiaoxia Jian, Xu He, Li Wang, Xiang Zhang, Peiyao Han

**Affiliations:** 1 School of Chemistry and Chemical Engineering, Nanjing University of Science and Technology, Nanjing, 10094 Jiangsu People’s Republic of China; 2 Xi’an Modern Chemistry Research Institute, Xi’an, 10065 Shaanxi People’s Republic of China

**Keywords:** Aluminum trihydride, Polydopamine, Morphological characterization, Interface properties, Polymer chemistry, Materials science

## Abstract

The reduction of interfacial interaction and the deterioration of processing properties of aluminum hydrides (AlH_3_) is the main challenges preventing its practical application. Here, a simple and effective core–shell structure aluminum hydrides@polydopamine (AlH_3_@PDA) complex was constructed through in-situ polymerization. The evolution of element states on the surface of AlH_3_ conducted by X-ray photoelectron spectroscopy indicated the successful introduction of PDA to form the core@shell structure, the thickness of the PDA coated layer increased with the increasing PDA dosage from 0.1 to 1.6% in mass fraction, and the maximum of thickness is 50 nm in TEM testing. Py GC/MS results proved that the increase of dopamine concentration leads to higher proportions of self-assemble units, whereas lower dopamine concentrations favor higher levels of chemical bonded components. Regarding whether PDA is a covalent polymer or a noncovalent aggregate of some species, the formation of intermediates, such as dopaminechrome and 5,6-dihydroxyindole played an important role to coordination interaction with AlH_3_ in FTIR, Raman, and UV–Vis spectra testing. Compared with pure AlH_3_, the formation of organic PDA coating improved AlH_3_ heat resistance. The adhesion work with GAP adhesive was also improved from 107.02 J/m^2^ of pure AlH_3_ to 111.13 mJ/m^2^ of AlH_3_@PDA-5 complex. This paper provides well support for further practical application of AlH_3_ in solid propellants.

## Introduction

Aluminum trihydride (AlH_3_) has also been taken into account as an ideal solid propellant fuel due to its high theoretical hydrogen storage capacity, its gravimetric and volumetric hydrogen capacities are 10.1% and 0.148 g/mL, respectively, which is twice as much as the value of liquid hydrogen (70.08 g/L). Compared with aluminum powder, it also has a higher calorific value of combustion^1,2^. However, aluminum trihydride is composed of two atoms with strong reduction, hence it has poor stability and is spontaneous decomposition in long-term storage at room temperature and the temperature range of solid propellant processing. Its incompatibility with other components and poor processability in the solid propellant remained unresolved.

At present, there are many ways to improve the thermal stability and compatibility of AlH_3_, including surface coating^3–6^, surface passivation^7^, and doping^8^, and so on, in addition to improving the crystallinity and purity of AlH_3_ by optimizing the synthesis and preparation conditions.

The polymer was a commonly used coating material for micro/nanoparticles, its long segment structure can not only achieve complete cover to the particle surface and complete physical isolation of particles but reduce the damage of the environment on it. In energetic materials, it also avoids direct contact among the components and improves compatibility. Schmidt et al.^9^ coated organic compounds containing nitrile on AlH_3_ to improve the compatibility with plasticizer components, such as trimethylolethane trinitrate. The results show the density of propellant containing AlH_3_ increased 26% through coating treatment, the free radicals on the surface of AlH_3_ are bonded with the nitrile group and passivate its surface, the compatibility between AlH_3_ and plasticizer has been greatly improved. Cai et al.^10^ coated fluoro rubber on the surface of AlH_3_ in supercritical fluid technology, the enthalpy of formation increases and Gibbs free energy decreases after coating, thus improving its thermal stability and effectively reducing electrostatic spark sensitivity of AlH_3_.

Since PDA was first reported as coating in 2007^11^, many follow-up works by other researchers using PDA as coating, bonding, or priming materials were reported^12,13^. The strong chemical adhesion to form the robust and compact core–shell structure and high rigidity of PDA enables it to provide great potential to efficiently reduce the sensitivity and improve the thermal stability and mechanical properties of energetic materials without a sacrifice of detonation power^14,15^.

PDA is reported in many energetic materials for cladding layer, such as 1,3,5-triamino-2,4,6-trinitrobenzene (TATB)^16^, 1,3,5,7-tetranitro-1,3,5,7-tetrazocane (HMX)^17,18^, and aluminum powder (Al)^19,20^. Zhang^21^ introduced PDA into AlH_3_ through simple situ polymerization. The coating retains its primary morphology, and the crystal form of AlH_3_ does not change after coating and the moisture absorption rate of AlH_3_ is greatly reduced after being coated. But the detailed morphological and structure changes and coating dosage are not reported. But the amount and structure of PDA coating are not disclosed, the mechanism of adhesion between AlH_3_ and PDA are also unknown. As an additive in the solid propellant to improve the specific impulse, its compatibility with other components in the propellant is an important parameter that directly affects its further application. In addition, the interfacial property of coated AlH_3_ and viscosity with the binder also effect its processing performance, all of these factors need to be investigated for future applications.

This study attempts to determine the effect of PDA coating on AlH_3_. Five different thickness PDA coated AlH_3_ composites were produced by in-situ polymerization. Compared to pristine AlH_3_, the microstructure, thermal properties, and surface morphology after PDA treatment were studied systematically. The effects of different PDA coatings on microstructural evolution and reaction mechanisms were analyzed and discussed. Then, compatibility and surface adhesion of AlH_3_ with glycidyl azide polymer (GAP) adhesive were performed to test the coating effect. This research may provide valuable guidance for the effects of AlH_3_ coating and the investigation of novel coating components.

## Results and discussion

### Microstructural features of AlH_3_@PDA composites

**Figure 1:**
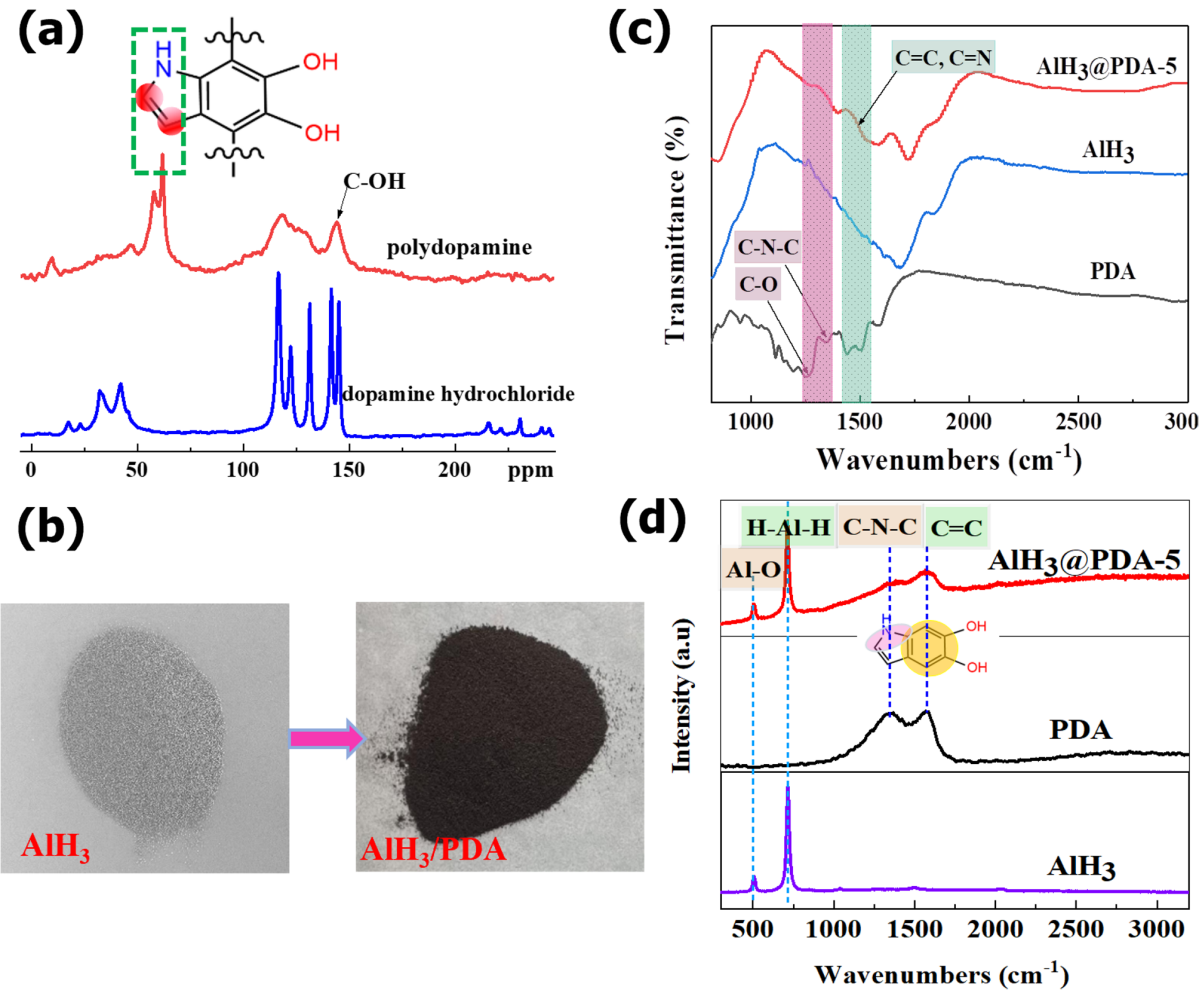
(**a**) ^13^C solid-state CP-MAS NMR spectra for dopamine hydrochloride and synthesized polydopamine. (**b**) photo of AlH_3_ and AlH_3_@PDA composites. (**c**) FT-IR patterns of AlH_3_ before and after PDA coating. (**d**) Raman patterns of AlH_3_ before and after PDA coating.

The ^13^C solid-state CP-MAS NMR spectra for dopamine hydrochloride and synthesized polydopamine are shown in Fig. 1a. The spectrum for dopamine hydrochloride contains peaks at 32.7 and 42.8 ppm for the aliphatic carbon atoms, as well as 116.6, 123.1, 131.7, 142.2, and 145.3 ppm for the aromatic carbon atoms. The PDA samples became gray with increasing polymerization time and gradually changed to thick brown in Fig. S1 in the Supporting Information. Accordingly the color of AlH_3_ particles changed from grey to thick brown of AlH_3_@PDA particles in Fig. 1b. After polymerization, the product peaks broaden due to the oligomeric nature of polydopamine and a new peak is observed downfield that correlates to quinone and 5,6-dihydroxyindole (DHI) formation that occurs due to oxidation of dopamine^22^.

In further FTIR testing in Fig. 1c, 1460, 1510, and 1596 cm^−1^ correspond to the characteristic peaks of C=C, C=N, and C=C (benzene ring) of PDA, respectively. While in Al@PDA-5 sample, the above three peaks become a large envelope peak, the C–O (phenolic hydroxyl) asymmetric stretching vibration peak at 1268 cm^−1^ remains unchanged, but the C–N–C characteristic peak at 1342 cm^−1^ shifts to high wave numbers, that means the characteristic structure of PDA is greatly affected by AlH_3_, and this effect on the internal indole ring is larger than hydroxyl end of PDA.

**Figure 2:**
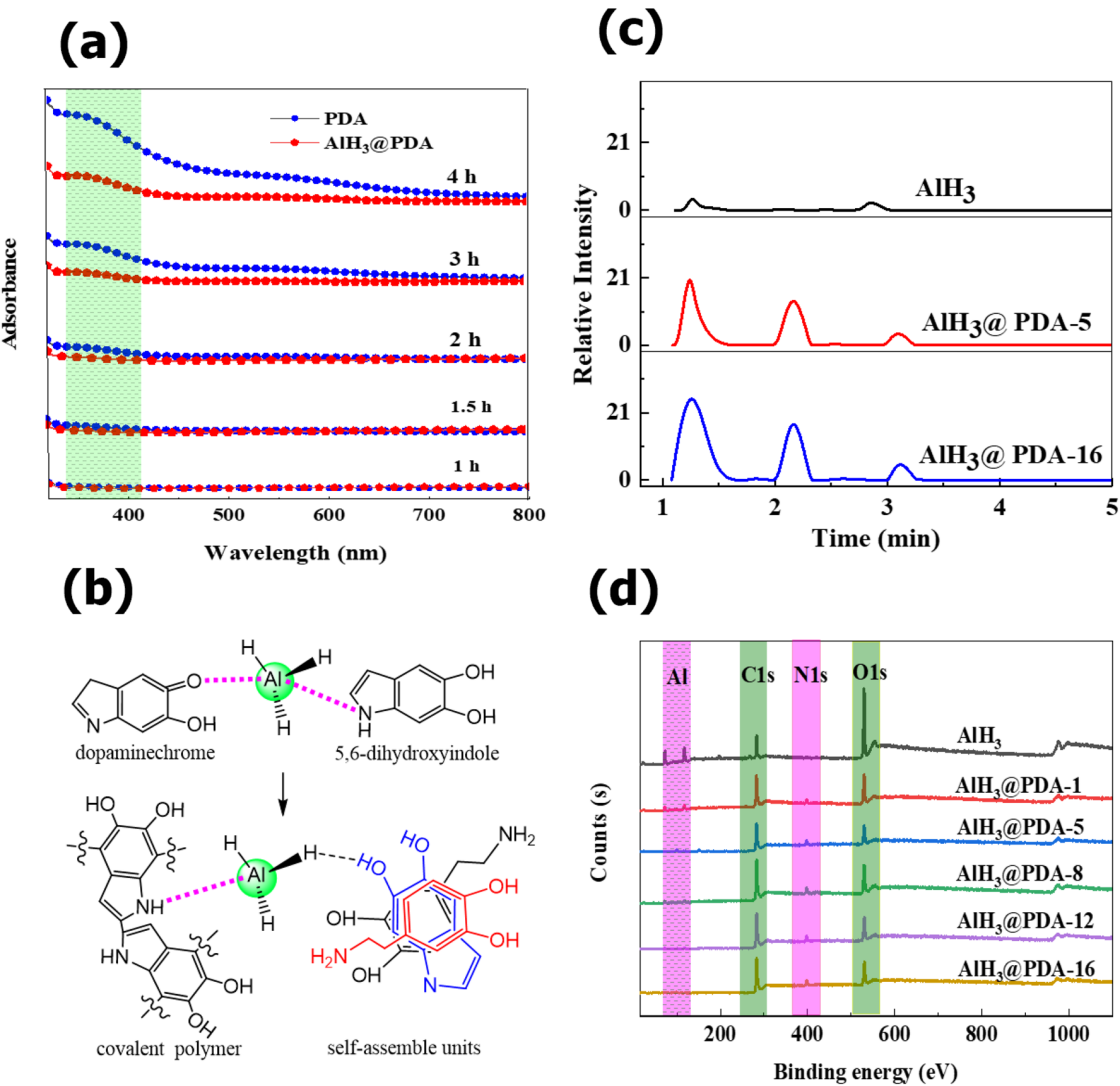
(**a**) UV–Vis patterns of PDA and AlH_3_@PDA at different polymerization time. (**b**) Schematic illustration for the coordination interaction between intermediate products and PDA with AlH_3_. (**c**) GC–MS chromatograms of AlH_3_@PDA composites. (**d**) X-ray photoelectron spectroscopy (XPS) of AlH_3_@PDA composites with different coating dosages.

Considering the lack of visible interaction between AlH_3_ and PDA in the FTIR spectrum, Raman testing was characterized in Fig. 1d. The peaks at 500 cm^−1^ and 717 cm^−1^ correspond to the Al-O and Al-H vibration peaks in aluminum hydride. In the spectrum of PDA, 1350 cm^−1^ and 1575 cm^−1^ correspond to the C–N–C peak and the C=C stretching vibration peak of the benzene ring. The characteristic peaks of both substances above are visible in the PDA@AlH_3_ complex. To be more special, the intensity of Al-H peak at 717 cm^−1^ decreases with the PDA content increases, indicating that AlH_3_ is becoming more and more fully coated (Fig. S2 in supporting information). The strength of C=C (benzene ring) peaks at 1575 cm^−1^ increases, while the strength of C–N–C peaks at 1350 cm^−1^ increases of PDA coating. This inconsistent growth is caused by two reasons. First, the proportion of chemical bonds in the coating system decreases, because mostly C–N–C bond belongs to chemical bonded PDA, and the self-assemble units increase with increased PDA content. Second, the interaction between the benzene ring of the coating layer PDA and AlH_3_. The shift of the peak at 1575 cm^−1^ towards the long wavelength was further verified, the positively charged aluminum atom of AlH_3_ reduces the density of the electron cloud of the benzene ring in DHI, the reduced energy to excite electrons eventually results in the absorption peak shifting to the long wavelength, that is, red shift occurs. Combined with the UV–vis absorbance, the spectrum of PDA and AlH_3_@PDA displays a shoulder at 363 nm in Fig. 2a, the forming capacities of DHI increase with extending the polymerization time, which are nevertheless different from the spectrum of PDA produced in the presence of AlH_3_, may be attributed to the presence of coordination interaction and hydrogen bonding between DHI, PDA and AlH_3_, this interaction were reported by other authors^23,24^. The corresponding intermediate products, such as dopaminechrome and 5,6-dihydroxyindole formed during the PDA in-situ coating process, their coordination interaction and hydrogen bond with AlH_3_ are marked in Fig. 2b.

Further inspecting the pyrolysis products of AlH_3_@PDA in Py GC/MS, as shown in Fig. 2c and Table S1 in the supporting information, we find that the products amount of self-assembled and covalent bond basically keep half of each other in AlH_3_@PDA-5, while in AlH_3_@PDA-16, almost all of the pyrolysis products are from self-assemble structures, this increased content of self-assemble with the increase of coating dosage further confirms our inference.

That is to say, polydopamine is not only covalent polymerization, but also has supermolecule aggregates (mainly composed of 5,6-dihydroxydihydroindole and its diketone derivative dopamine pigment), which are bound together through the combination of charge transfer, π stacking and hydrogen bond interaction^15,25^. In our experimental process, increasing dopamine concentration will lead to a higher proportion of DHI containing units, and polydopamine is most commonly represented as an aggregation of oligomers, where monomer units are connected through self-assembly. As a result, the composites form a much stronger interaction.

### Surface properties of AlH_3_@PDA composites

Figure 2d and Table S2 in the supporting information show the high-resolution XPS spectra of C1s, N1s, and O1s peaks for AlH_3_, and AlH_3_@PDA composites with different PDA contents. The surface of pure AlH_3_ only contains three elements: Al, C, and O. After PDA coating, the surface of AlH_3_@PDA composites contains not only three elements: Al, C, and O but also the characteristic N element peak of the polydopamine film. The content of N increased from 0 to 5.8% and Al suffered from an obvious decrease from 43.8 to 1.0% for pure AlH_3_ to AlH_3_@PDA-16 composite, while the N/Al ratio also changed from 0 to 5.8, which confirmed that the coating content of PDA on the surface of AlH_3_ crystals increased. The N/C ratio keeps stable (~ 0.07–0.08) with the increasing coating weight, which means PDA is mostly coated in the surface of AlH_3_.

**Figure 3:**
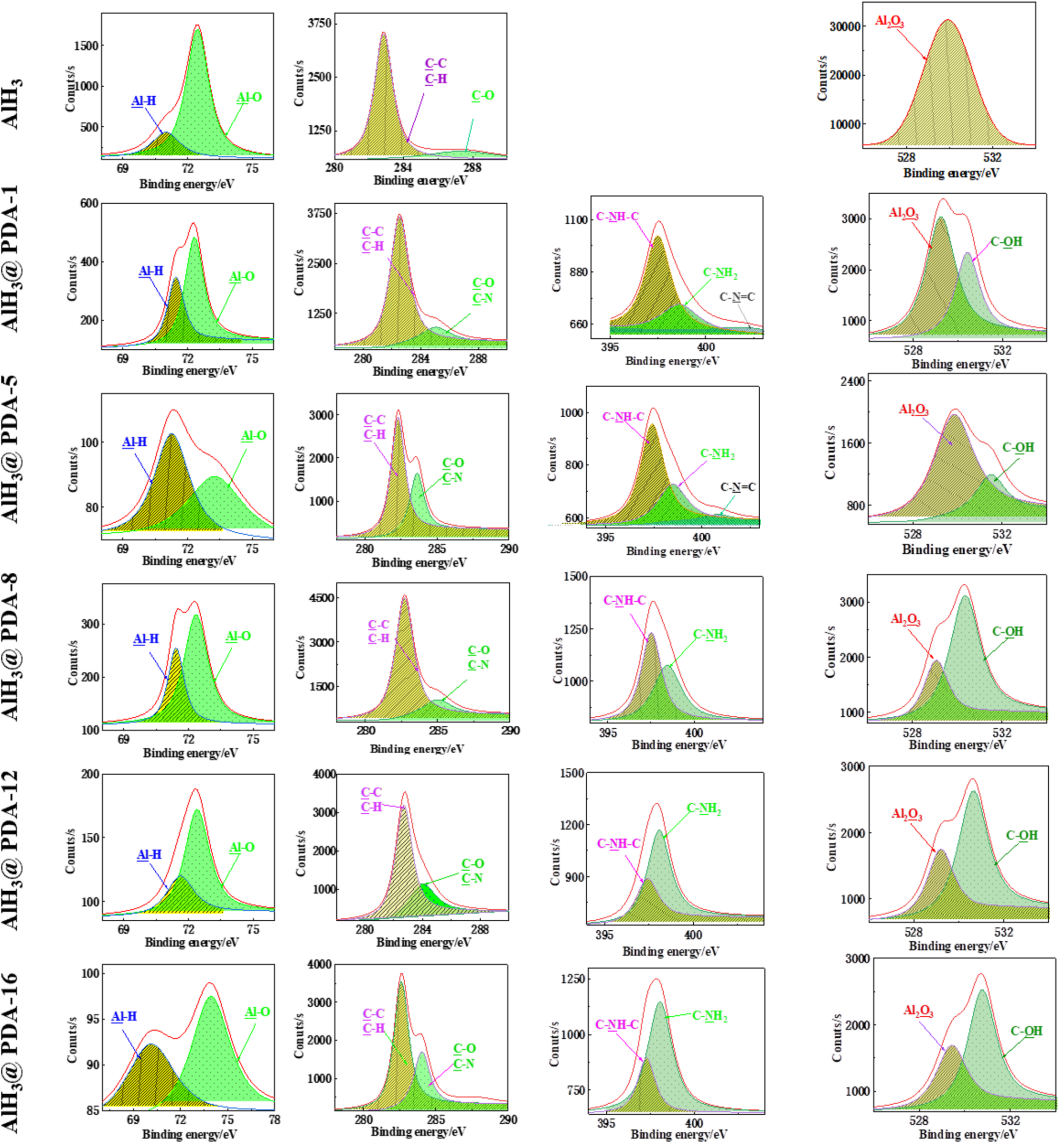
High-resolution XPS patterns of AlH_3_@PDA with different coating dosages.

The chemical compositional information of polymer shell layer could be obtained in XPS spectra. The decreasing peaks intensities of Al-H and Al-O at 72.5 eV in Al spectra with PDA increasing confirmed the successful coating of PDA on the surface of AlH_3_ crystals. The peak belonged to C–C/C–H and C–O/C–N at about 283.3 eV and 284.2 eV in the C1s spectra, respectively. Regardless of self-assembled structure or covalent bond of PDA, its C–C, C–H, and C–O mostly come from the contribution of the benzene ring, so the peak strength did not undergo significant changes. The new peaks ascribed to C–NH–C and C–NH_2_ at about 397.5 and 398.1 eV in the N1s spectra. The peak at 529.1 eV comes from Al_2_O_3_ in the O1s spectra, that’s mean the surface of AlH_3_ is partially oxidized. And the peak intensity of the uncoated AlH_3_ surface is the higher than AlH_3_@PDA composites, indicating that polydopamine coating can prevent oxidation of AlH_3_. Especially, the C–NH_2_ peak intensity at 398.1 eV in N1s and the C–OH peak at about 530.6 eV in O1s increase with the increase of PDA coating amount, that is, the proportion of self-assembly structures increased in the coating system (Fig. 3).

### Morphological characterization of AlH_3_@PDA composites

**Figure 4:**
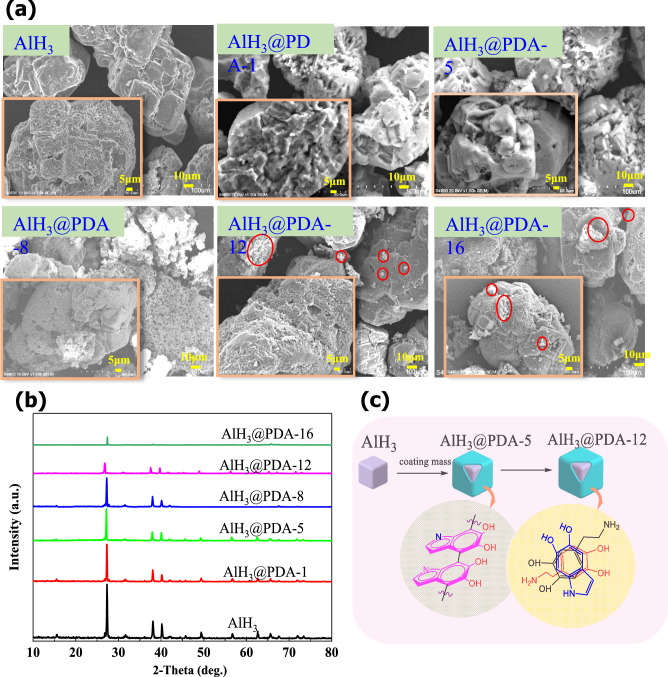
(**a**) SEM micrographs of AlH_3_ with different PDA dosages. (**b**) XRD patterns of AlH_3_@PDA with different coating dosages. (**c**) PDA structure on the AlH_3_ surface at different concentration.

Combined with SEM images in Fig. 4a, pure AlH_3_ particles are crystals with clear edges and corners. The surface morphologies of the AlH_3_ crystals exhibited ignorable change after PDA modification. And the sharp edges in the AlH_3_ crystal become less and less with the increase of the coating dosage, which is beneficial to reduce its sensitivity. And the white AlH_3_ crystals gradually changed from gray to thick brown with increasing PDA coating amount, it may be attributable to the difference in surface features and specific surface area. The partial enlargement image clearly shows that the surface of AlH_3_ is covered by PDA coating. The coating thickness is uneven although AlH_3_ is tightly bound by the PDA coating, the aggregation of PDA coating becomes more and more serious with further coating of PDA, as shown by the red circles in Fig. 4a, which is unfavorable to the energy performance of AlH_3_. Therefore, the actual coverage of PDA should be less than 1.2% (mass fraction).

In Fig. 4b, all diffraction peaks can be indexed, 2θ = 27.1°, 37.9°, 40.0°, 45.6°, 49.2°, 56.6°, 62.5°, 65.5°, and 67.5°correspond to (012), (111), (110), (006), (202), (024), (116), (122), and (018) crystal planes of AlH_3_, respectively. The presence of impurity peaks at 15.3° and 31.4° may originate from the aluminum oxide produced by partially oxidized aluminum trihydride powder. Compared with cell parameters of AlH_3_ (a = b = 4.4565 Å, c = 11.826 Å; α = 90°, β = 90°, γ = 120°) and AlH_3_@PDA (a = b = 4.4578 Å, c = 11.829 Å; α = 90°, β = 90°, γ = 120°), there was no significant change, indicating that the crystal nature of AlH_3_ coated with polydopamine did not change.

Previous research work has shown that the catechol groups in PDA are capable of forming hydrogen bonds, metal–ligand complexes, and quinhydrone charge-transfer complexes, which afford strong adhesion to various types of materials^26,27^. Although catechol groups are present in PDA, the blend of polymerized PDA and AlH_3_ is unable to bond well together in our study. The peak of the C=C absorption peak in FT-IR moved from 1680 cm^−1^ of PDA and AlH_3_ blend to 1670 cm^−1^ of AlH_3_@PDA, and the intensity of the peak significantly increased. Furthermore, the peak of AlH_3_@PDA composite is higher than the PDA and AlH_3_ blend in the UV spectrum as shown in Figs. S3 and S4 in the supporting information, although AlH_3_ can release hydrogen gas and is partly oxidized to Al_2_O_3_, but Al_2_O_3_ hardly interacts with PDA. These are related to the interaction between intermediate products and AlH_3_ in the PDA polymerization process.

AIH_3_ as a lewis acid can interact with electron donor lewis bases to form dynamically and thermodynamically stable AlH_3_ complexes, with Al as the center and coordination numbers of 4 or 5^23^. And the lewis base ligands include amine, phosphine, carbene, ether, and other compounds^24,28–30^. In our work, dopaminechrome and 5,6-dihydroxyindole generated during the PDA polymerization process can act as a ligand with nitrogen and oxygen donor sites.

Based on above results and Py GC/MS testing (Fig. 2c and Table S1 in the supporting information), we infer that the structure of AlH_3_@PDA is shown in Fig. 4c, that is covalent bond and self-assembly are the main components in AlH_3_@PDA-5 and AlH_3_@PDA-16, respectively.

**Figure 5:**
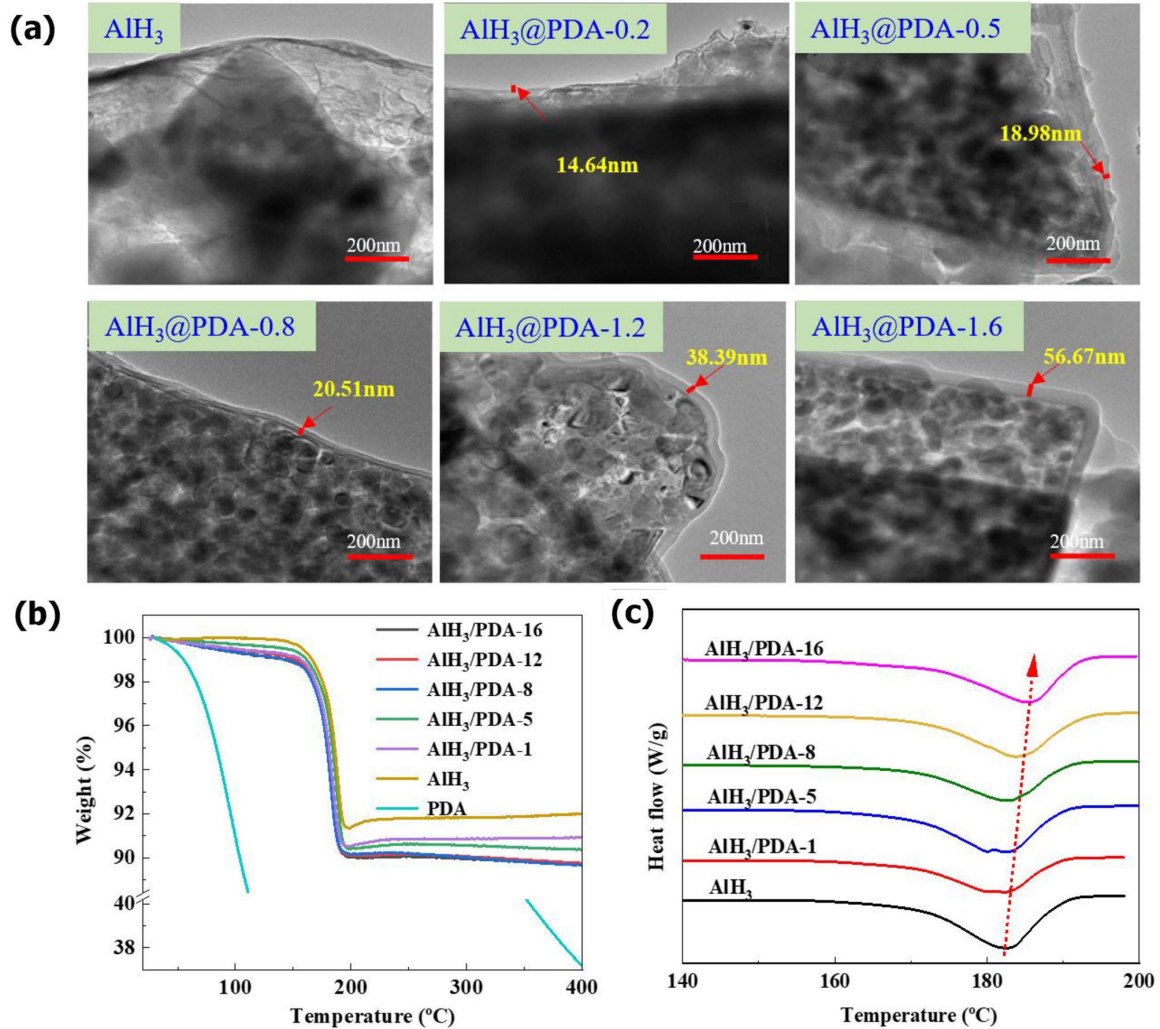
(**a**) TEM image of AlH_3_@PDA composites. (**b**) TGA curves of AlH_3_@PDA composites. (**c**) DSC curves of AlH_3_@PDA composites.

To obtain detailed information on the coating layer, we further measured the PDA thickness of different dosages in TEM. As illustrated in Fig. 5a, the inner dark zones between AlH_3_ particle and the PDA layer, which is the oxide cap of the AlH_3_ particle, remains constant across all testing PDA coating dosage. The white zones of the AlH_3_ surface were uniform PDA coating layer and the thickness of the coated layer increased with the PDA dosage, which are 14.63, 18.98, 20.51, 24.39, and 66.67 nm (Al_2_O_3_ layer are also included), respectively. However, the difference in the thickness of AlH_3_@PDA-12 and AlH_3_@PDA-16 is relatively small, indicating that the coating dosage of dopamine has a maximum value, of about 50 nm, which is consistent with the reported value^31^.

### Thermal properties of AlH_3_@PDA composites

The mass loss in TGA testing showed a maximum of 9.12% (mass fraction) of AlH_3_ sample in Figs. 5b and S5 in the supporting information, which means the escaped hydrogen in the material, was close to the theoretical hydrogen content of 10.1%. Although the mass loss of AlH_3_@PDA composites increased with the coating dosage, the overall weight loss was still less than the theoretical 10.1% of AlH_3_. As an additive in the solid propellant to improve the specific impulse, the geometry of the AlH_3_ crystal surface played a crucial role in determining the interfacial interactions with other components in solid propellant. The difference of coverage degree of AlH_3_@PDA composites in Fig. 5a, could be used to better understand the relationship between the coating structure and thermal phase transition properties. Figure 5c illustrates the changes that occur during heating for pristine AlH_3_ and PDA-treated AlH_3_. An endothermic peak of pristine AlH_3_ was observed at 181.6 °C, which is in accordance with the endothermic reaction being attributed to the dehydriding of AlH_3_^32,33^. With PDA coating, the transition temperature slightly increased to 185.6 °C. The further coating with PDA made the transition peak shift to higher temperatures, indicating that the thermal stability of AlH_3_ is greatly improved after PDA modification, which is due to the formation of organic PDA to improve its heat resistance.

### Interface properties of AlH_3_ and GAP adhesive

**Figure 6:**
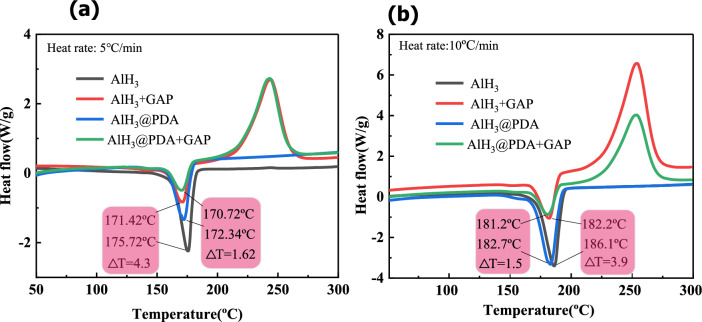
DSC curves of AlH_3_ and AlH_3_-GAP mixture before and after PDA coating at the heating rates of 5 °C/min (**a**) and 10 °C/min (**b**).

Compatibility is the ability of materials to resist chemical changes when they interact with other materials, both the compatibility and interaction between energetic materials are of great importance. Differential scanning calorimetry (DSC) at different heating rates was performed to investigate the compatibility and the interaction between the AlH_3_ and GAP adhesive as shown in Fig. 6, and the criteria used to judge compatibility were based on the standardization agreement STANAG 4147^34^. The other heating rate results were homologous as shown in Table S3 in the Supporting Information. The exothermic peak difference (ΔT) of AlH_3_ are 4.3 and 3.9 at the heating rate of 5 °C/min and 10 °C/min. According to the evaluated standards of compatibility, they are the fair compatibility between the two materials. While in AlH_3_@PDA-5/GAP mixture, the exothermic peak difference (ΔT) of AlH_3_@PDA are 1.6 and 1.5 at the heating rate of 5 °C/min and 10 °C/min, that means coated AlH_3_@PDA particles changed to compatible from incompatible with AlH_3_ with GAP adhesive.

**Figure 7:**
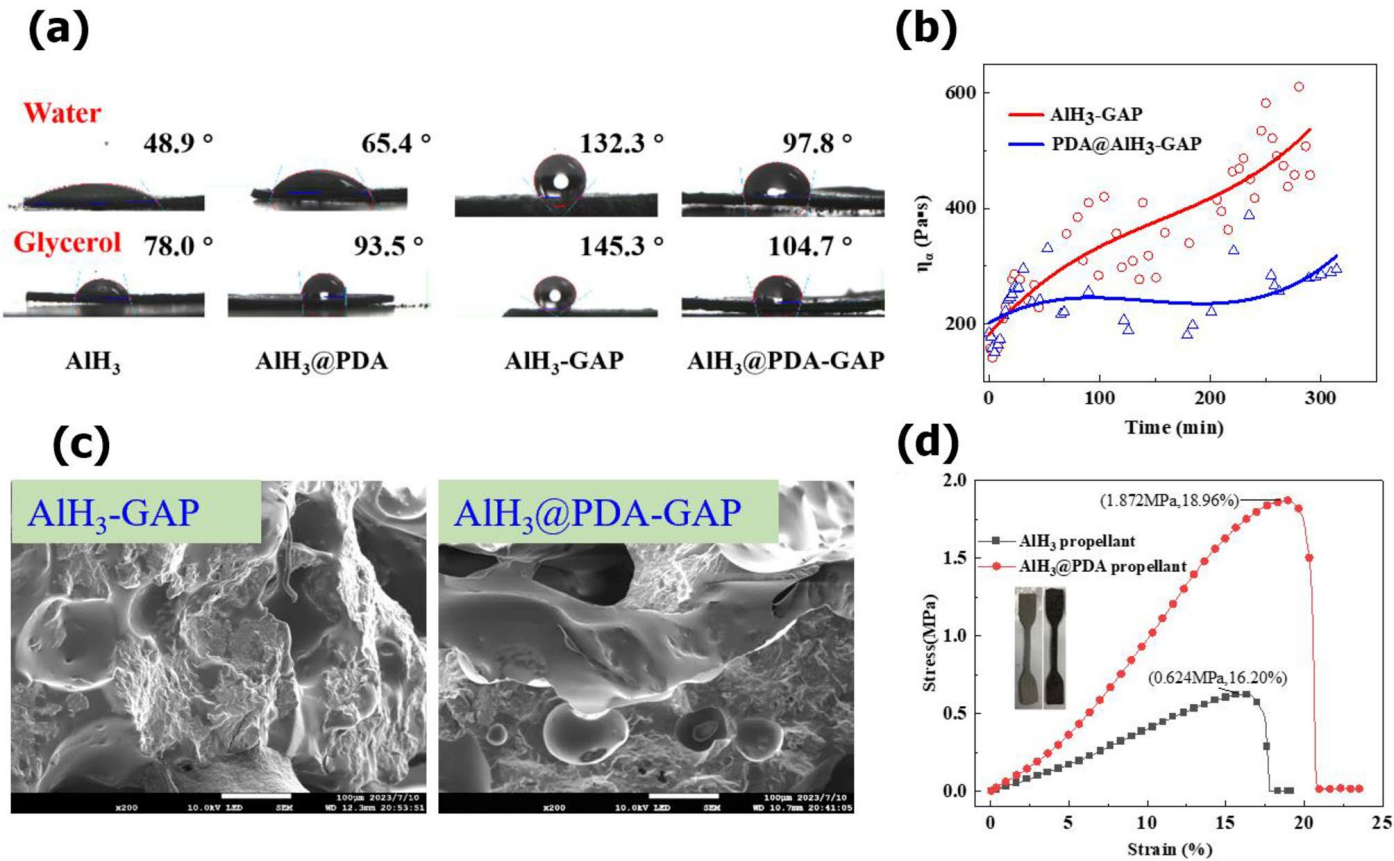
(**a**) Typical images of the contact angle on the surface of AlH_3_ and AlH_3_ with GAP before and after PDA coating. (**b**) The relationship between viscosity and time of AlH_3_ and GAP compound. (**c**) SEM images of AlH_3_-GAP and AlH_3_@PDA-GAP compound. (**d**) The mechanical properties of AlH_3_ based propellants.

The contact angle images of different particles are shown in Fig. 7a and S6 in the supporting information. The interfacial energy and adhesion work are calculated to analyze the interfacial properties of AlH_3_ before and after PDA coating in Table 1.

**Table 1:** Contact angles of AlH_3_ crystals, surface energies, and adhesive work between AlH_3_ and GAP.

Sample	Contact angle (deg)		Surface energy (mN/m)			Adhesive work (mJ/m^2^)
	water	glycerol	$\gamma_{s}^{p}$	$\gamma_{s}^{d}$	$\gamma_{Ls}^{{}}$	
AlH_3_	48.9	78.0	5.76	41.98	10.73	90.84
AlH_3_/GAP	132.3	145.3	12.41	47.55	6.78	107.02
AlH_3_@PDA-5	65.4	93.5	7.47	55.10	12.51	103.89
AlH_3_@PDA-5/GAP	97.8	104.7	12.87	52.37	7.94	111.13

Based on the Young-Dupre equation^35,36^, the relationship between surface energy, contact angle ($\theta$), and adhesion work can be expressed as follows:

1 $${\upgamma }_{LS} = {\upgamma }_{S} - {\upgamma }_{L} cos\theta$$

2 $${\text{W}}_{a} = {\upgamma }_{L} + {\upgamma }_{S} - {\upgamma }_{LS} = {\upgamma }_{L} 1 + cos\theta$$

where ${\upgamma }_{S}$, ${\upgamma }_{L}$ and ${\upgamma }_{LS}$ are the surface energy of solid, liquid, and solid–liquid interfaces, respectively. ${\text{W}}_{a}$ is the adhesion work of solid–liquid interface.

According to the surface energy component method of Owens and Wendt, the solid–liquid interfacial energy can be expressed as:

3 $${\upgamma }_{LS} = {\upgamma }_{S} + {\upgamma }_{L} - 2\sqrt {\gamma_{S}^{p} \gamma_{L}^{p} } - 2\sqrt {\gamma_{S}^{d} \gamma_{L}^{d} }$$

where $\gamma_{S}^{p}$ and $\gamma_{L}^{p}$ are the polar components of solid and liquid surface energy, respectively. $\gamma_{L}^{d}$ and $\gamma_{L}^{d}$ are nonpolar components. So

4 $${\upgamma }_{S} = \gamma_{S}^{p} + \gamma_{S}^{d}$$

5 $${\upgamma }_{L} = \gamma_{L}^{p} + \gamma_{L}^{d}$$

Based on Eqs. (1) and (3), Eq. (6) can be obtained:

6 $${\upgamma }_{L} \left( {cos\theta + 1} \right) = 2\sqrt {\gamma_{S}^{p} \gamma_{L}^{p} } + 2\sqrt {\gamma_{S}^{d} \gamma_{L}^{d} }$$

Based on the contact angles of different droplets on AlH_3_ before and after coating, the surface energy components, including the polar component $\gamma_{S}^{p}$ and dispersion component $\gamma_{S}^{d}$ were calculated by Owens and Wendt methods.

According to Eq. (6), two kinds of solvents were used to determine the surface energy of AlH_3_ crystal before and after coating. The solvents used in this experiment are glycerol and water, in which the $\gamma_{L}^{p}$ and $\gamma_{L}^{d}$ of glycerol are 21.7 and 42 mJ/m^2^ respectively, and the $\gamma_{L}^{p}$ and $\gamma_{L}^{d}$ of water are 51.7 and 21.1 mJ/m^2^ respectively. The calculated results obtained from Eqs. (1), (2), and (6) are shown in Table 1.

The surface energy of the AlH_3_ is 10.73 mJ/m^2^. After PDA coating, the surface energy of AlH_3_@PDA-5 is improved to 12.51 mJ/m^2^. This improvement of the surface energy is mainly due to the increase of the nonpolar PDA component. The adhesion work is used to measure the energy required for interface separation, which can indicate the bond strength of the solid–liquid interface. Compared with the pure AlH_3_, the adhesion work improved from 90.84 mJ/m^2^ of AlH_3_ to 103.89 mJ/m^2^ of AlH_3_@PDA-5. This is related to PDA forming covalent and non-covalent bonds with strong adhesion on the surface of AlH_3_.

Further calculated interfacial energy and adhesion work of AlH_3_ with GAP adhesive, they are 7.94 mN/m and 111.13 mJ/m^2^ to AlH_3_@PDA-5 with GAP adhesive, which are higher 17.1% and 3.84% than those of the uncoated AlH_3_ with GAP adhesive. This indicates that the interfacial interaction and interfacial bond strength between coated AlH_3_ and GAP are significantly improved. The morphology of the filler surface was one of the primary factors which influenced the interactions at the interface of polymer matrix-filler particle. This was also demonstrated in reduced viscosity during the mixing process in Fig. 7b and morphology after mixing AlH_3_, GAP, and diphenyl methane diisocyanate (MDI) in Fig. S7 in the supporting information.

There were so many pores on the fractured surface of the compound in SEM morphology, which resulted from the AlH_3_ detachment caused by the brittle fracture process in Fig. 7c. However, compared with AlH_3_ and GAP compound, the system of AlH_3_@PDA-5 with GAP adhesive displayed smooth fractured surfaces because of the reduced interfacial interaction of AlH_3_/GAP, that will be beneficial to improve the processability and mechanical property of the AlH_3_ in high energy solid propellant. The tensile stress increased from 0.62 MPa of AlH_3_ based propellant to 1.87 MPa of PDA coated AlH_3_ based propellant in Fig. 7d.

## Conclusion

In summary, we have reported a core–shell structured AlH_3_@PDA energetic microspheres intending to improve the interfacial interaction and processing performance with the azide GAP adhesive. Their structure and morphology, thermal behavior, interfacial behavior, and function mechanism were studied under different PDA coating conditions. The experimental investigations revealed that PDA was successfully generated on the surface of AlH_3_ by in situ-polymerization, and PDA interfacial coating on the surface of AlH_3_ is positively correlated with the concentration of mixed solution of dopamine hydrochloride. The thickness of coated PDA increased simultaneously, and it reached a peak value of 50 nm with 1.6% PDA content in mass fraction. The proportion of multilayer self-assembly units, compared to covalently-linked PDA, also increased with increased PDA content. We also demonstrated the dominant role of intermediate products, such as dopaminechrome and 5,6-dihydroxyindole formed during the PDA in-situ coating process, their coordination interaction and hydrogen bond with AlH_3_ suffered from the robust adhesion. The formation of organic PDA coating improved AlH_3_ heat resistance, the thermal loss temperature enhanced with increased PDA dosages, and the mass loss of AlH_3_@PDA composites are close to the theoretical value (10.1%) of pure AlH_3_. Further calculated interfacial energy and adhesion work of PDA coated AlH_3_ with GAP adhesive are higher than those of the uncoated AlH_3_ with GAP adhesive. That means the interfacial interaction between coated AlH_3_ and GAP are significantly improved. This research provides new thought for enhanced interfacial interaction of AlH_3_ and its application in solid propellants.

## Materials and methods

### Materials

The AlH_3_ powders (purity > 99.9%) with an average particle size of 200 μm were provided by Xi’an modern chemistry research institute, Dopamine hydrochloride was purchased from Sigma-Aldrich Co., Ltd. Tris hydrochloric acid buffer solution(pH = 7.5–8.5) and anhydrous ethanol were obtained from Aladdin Biochemical Co., Ltd of Shanghai. GAP was obtained from the Shanghai space propulsion technology research institute.

### Preparation of core–shell structured AlH_3_@polydopamine energetic microspheres

AlH_3_ particle was first dispersed to anhydrous ethanol, then it was added to the mixed solution of dopamine hydrochloride and Tris hydrochloric acid buffer solution, and the pH value of the resulting solution was adjusted to 8.5 using 40% ammonium hydroxide. The reaction was continuous up to 24 h under string in room temperature. The polymerization of dopamine can be observed by the color of the solution changed from white to dark brown. The obtained dark brown solution was filtered. The samples were rinsed with anhydrous ethanol and dried in a vacuum oven. The experimental operation process is shown in Fig. 1. The mass ratios of dopamine to AlH_3_ are 0.1%, 0.5%, 0.8%, 1.2%, and 1.6% in mass fraction, which were called AlH_3_@PDA-1, AlH_3_@PDA-5, AlH_3_@PDA-8, AlH_3_@PDA-12, and AlH_3_@PDA-16, respectively.

### Characterization

Structural characterization of the AlH_3_ and PDA@AlH_3_ samples was performed by SSNMR-solid-state NMR spectrometer (AVANCE III WB 400 MHz, 13C spectra), Fourier infrared spectrometer (FT-IR, NicoletiS10, the scan range is 4000 cm^−1^–525 cm^−1^), microscopic confocal Raman spectrometer (Raman, INVIA, origin distance from the surface plasmon resonance is 785 nm), UV–visible spectrophotometer (EVOLUTION220, wavelength range 190–1100 nm) and high temperature thermal lysis-gas chromatography-mass spectrometry (Py GC/MS, 2-EGC/PY-3030D, by temperature-programmed at 50–500 °C, heating rate in 20 K/min, splitting decomposition 20 s). The morphology of the samples was examined by field-emission scanning electron microscopy (SEM, JSM-7800F PRIME), high-power transmission electron microscopy (TEM, JEM 2100f) and X-ray diffractometer (XRD, ADVANCE D8, Cu Ka radiation, λ = 1.5418 A). Surface chemical element was analyzed using X-ray elemental analysis (XPS, GENESIS XM, Equipped with monochrome AlKa radiation (1486.6 eV)). The thermal analysis and compatibility were studied by TGA (TGA/SDTA851E, N_2_:50 mL/min, the temperature range is 50–400 °C, heating rate of 10 K/min) and differential scanning calorimeter (DSC, DSC823E, N_2_:50 mL/min, the temperature range is 50–300 °C, heating rate of 10 K/min). Surface adhesion properties were measured and calculated by an optical contact angle measuring instrument (Kruss DSA100), the interface energy and adhesion work were calculated using the Young-Dupre equation.

### Supplementary Information

Supplementary Information.

## Data Availability

All data generated or analysized during this study are included in this published article and its supplementary information files.
