# Supplementary material for: Microstructure and interaction in aluminum hydrides@polydopamine composites and interfacial improvement with GAP adhesive
Source: Sci Rep. 2024 May 1;14:10013. doi: 10.1038/s41598-024-59944-1 (PMC11063057; doi:10.1038/s41598-024-59944-1)
Supplement: Supplementary file 1 — Supplementary Information. [file 41598_2024_59944_MOESM1_ESM.docx]

## Supporting Information

**Microstructure and interaction in aluminum hydrides@polydopamine composites and interfacial improvement with GAP adhesive**

An Qi^1^, Xie Wuxi^2^ , Li Yajin^2^ , Jian Xiaoxia^1*^ , He Xu^1^, Wang Li^1^ , Zhang Xiang^1^, Han Peiyao^1^

1. School of Chemistry and Chemical Engineering, Nanjing University of Science and Technology, Nanjing 210094, Jiangsu, People’s Republic of China

2. Xi’an Modern Chemistry Research Institute, Xi’an 710065, Shaanxi, People’s Republic of China.

**Corresponding Author**

* E-mail: [jxx259@njust.edu.cn](mailto:jxx259@njust.edu.cn) (Xiaoxia Jian)

**1. Sample Preparation**

AlH_3_ crystal (1 g) was added into the 20 mL anhydrous ethanol and followed by sonication for 30 min, while dopamine hydrochloride (0.5 g) was dissolved in 20 mL Tris HCl buffer solution. After 30min, AlH_3_ dispersion was added to the dopamine solution. Then ethanol (10 mL), ammonium hydroxide (0.6 mL), and 18 MΩ DI water (10 mL) were mixed in a separate beaker for 30 min under ambient conditions. The dopamine solution was further added to the water-alcohol mixture, covered with aluminum foil, and left to stir for 24 h at room temperature. The mixture was then centrifuged at 10 000 rpm for 30 min and the supernatant was disposed of and the precipitate was collected. The collected precipitate was redispersed in anhydrous ethanol to remove residual ammonium hydroxide and unreacted dopamine monomer. The synthesized AlH_3_@polydopamine composite was then dried in a 30 °C oven.

**2. Supplemented Figures and Tables**


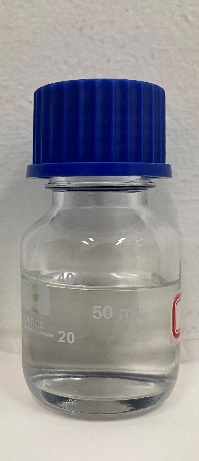

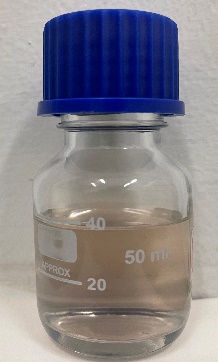

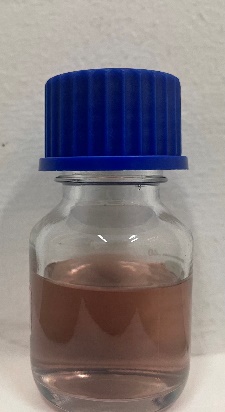

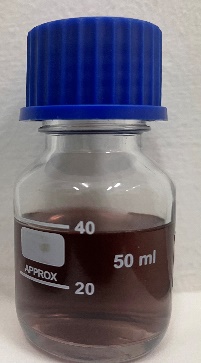

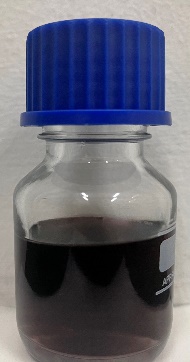

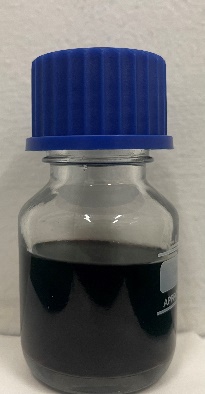


0 h

1 h

2 h

3 h

5 h

6 h

**Figure S1**. Photos of AlH_3_@PDA with different PDA coating time.

**Figure S2**. Raman spectrum of AlH_3_@PDA with different coating dosages.

**Figure S3.** FT-IR spectrum of AlH_3_@PDA, AlH_3_/PDA blend, and Al_2_O_3_@PDA.

**Figure S4.** UV spectrum of AlH_3_@PDA, AlH_3_/PDA blend, and Al_2_O_3_@PDA.

**Figure S5**. TG curves for uncoated AlH_3._

**Figure S6**. Contact angle of AlH_3_ before and after PDA coating.


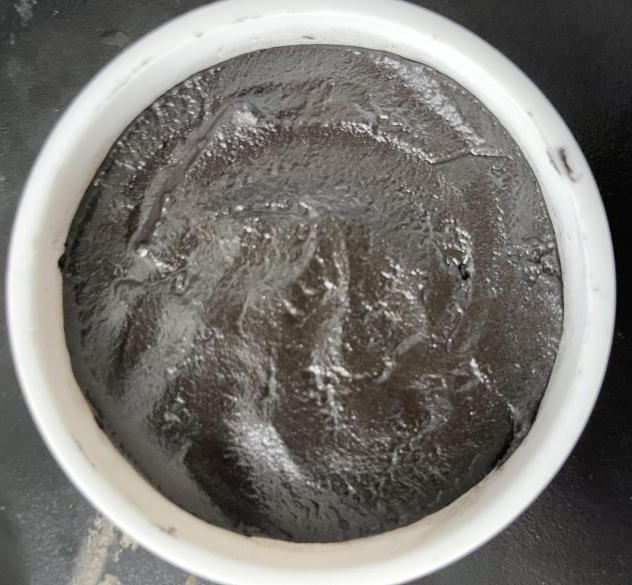


AlH_3_@PDA-GAP


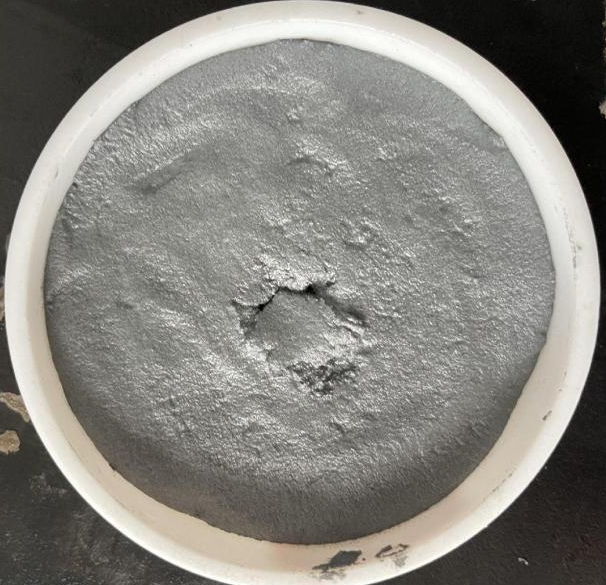


AlH_3_-GAP

**Figure S7**. Photos of blends of AlH_3_-GAP and AlH_3_@PDA-GAP.

**Table S1**. Main components identified by Py-GC/MS

| Retention time /min | Peak Area of samples/% | | | Identification |
| --- | --- | --- | --- | --- |
|  | AlH_3_ | AlH_3_@PDA-5 | AlH_3_@PDA-16 |  |
| 1.25 | 0.96 | 15.71 | 36.69 | CH_3_CH(NH_2_)CH_2_COOH |
| 1.29 | - | 14.15 | - | CH_3_NHCH_2_COOH |
| 1.45 | - | 2.78 | 5.04 | CH_3_CH_2_OCH_2_CH_3_ |
| 2.16 | - | 28.65 | 31.29 | N(CH_2_CH_3_)_3_ |
| 3.11 | 2.64 | 13.37 | 17.71 | C_6_H_5_(CH_3_) |

**Table S2.** Elemental content of AlH_3_ and AlH_3_@PDA of XPS

| Element | AlH_3_ | AlH_3_@PDA-1 | AlH_3_@PDA-5 | AlH_3_@PDA-8 | AlH_3_@PDA-12 | AlH_3_@PDA-16 |
| --- | --- | --- | --- | --- | --- | --- |
| C | 19.12 | 59.2 | 66.6 | 68.1 | 68.4 | 73.1 |
| O | 37.08 | 30.1 | 25.1 | 23.6 | 23.5 | 20 |
| N | 0 | 3.9 | 4.5 | 5.1 | 5.7 | 5.8 |
| Al | 43.8 | 6.8 | 3.8 | 3.2 | 2.4 | 1.0 |
| N/C | 0 | 0.07 | 0.07 | 0.07 | 0.08 | 0.08 |
| N/Al | 0 | 0.57 | 1.18 | 1.59 | 2.38 | 5.80 |

**Table S3.** The evaluated standards of compatibility for energetic materials.

| Heating rate/ °C·min^-1^ | Prue AlH_3_ | | | AlH_3_@PDA-5 | | | |
| --- | --- | --- | --- | --- | --- | --- | --- |
|  | T_p1_ | T_p1’_ | △T_1_ | | T_p2_ | T_p2’_ | △T_2_ |
| 1 | 166.72 | 163.56 | 3.16 | | 163.01 | 161.09 | 1.92 |
| 5 | 174.97 | 171.30 | 3.67 | | 172.30 | 170.58 | 1.72 |
| 10 | 186.04 | 182.00 | 4.04 | | 182.83 | 181.25 | 1.58 |
| 20 | 197.33 | 191.87 | 5.46 | | 191.27 | 189.83 | 1.44 |

The maximum exothermic peak temperature of AlH_3_ was expressed by the variable T_p1_, while the maximum exothermic peak temperature of the mixture (weight ratio of AlH_3_ with GAP is equal to 1:1) was expressed by the variable T_p1’_. The difference between the T_p1_ and T_p1’_ was calculated by ΔT_1_ = T_p1_– T_p1’_. The maximum exothermic peak temperature of AlH_3_@PDA-5 was expressed by the variable T_p2_, while the maximum exothermic peak temperature of the mixture (weight ratio of AlH_3_@PDA-5 with GAP is equal to 1:1) was expressed by the variable T_p2’_. The difference between T_p2_ and T_p2’_ was calculated by ΔT_2_ = T_p2_ - T_p2’_.
